# Quantitative Assessment of Relationship between Population Exposure to PM_2.5_ and Socio-Economic Factors at Multiple Spatial Scales over Mainland China

**DOI:** 10.3390/ijerph15092058

**Published:** 2018-09-19

**Authors:** Ling Yao, Changchun Huang, Wenlong Jing, Xiafang Yue, Yuyue Xu

**Affiliations:** 1 Guangdong Open Laboratory of Geospatial Information Technology and Application, Key Laboratory of Guangdong for Utilization of Remote Sensing and Geographical Information System, Engineering Technology Center of Remote Sensing Big Data Application of Guangdong Province, Guangzhou Institute of Geography, Guangzhou 510070, China; yaoling@lreis.ac.cn (L.Y.); jingwl@lreis.ac.cn (W.J.); 2 State Key Laboratory of Resources and Environmental Information System, Institute of Geographic Sciences and Natural Resources Research, Chinese Academy of Sciences, Beijing 100101, China; lexf@lreis.ac.cn; 3 Jiangsu Center for Collaborative Innovation in Geographical Information Resource Development and Application, Nanjing Normal University, Nanjing 210023, China; 4 School of Geography Science, Nanjing Normal University, Nanjing 210023, China; huangchangchun@njnu.edu.cn; 5 School of Geography and Ocean Science, Nanjing University, Nanjing, 210023, China; 6 Jiangsu Provincial Key Laboratory of Geographic Information Science and Technology, Nanjing University, Nanjing 210023, China

**Keywords:** spatial heterogeneity, population exposure, economic effects, quantitative analysis

## Abstract

Analyzing the association between fine particulate matter (PM_2.5_) pollution and socio-economic factors has become a major concern in public health. Since traditional analysis methods (such as correlation analysis and geographically weighted regression) cannot provide a full assessment of this relationship, the quantile regression method was applied to overcome such a limitation at different spatial scales in this study. The results indicated that merely 3% of the population and 2% of the Gross Domestic Product (GDP) occurred under an annually mean value of 35 μg/m^3^ in mainland China, and the highest population exposure to PM_2.5_ was located in a lesser-known city named Dazhou in 2014. The analysis results at three spatial scales (grid-level, county-level, and city-level) demonstrated that the grid-level was the optimal spatial scale for analysis of socio-economic effects on exposure due to its tiny uncertainty, and the population exposure to PM_2.5_ was positively related to GDP. An apparent upward trend of population exposure to PM_2.5_ emerged at the 80th percentile GDP. For a 10 thousand yuan rise in GDP, population exposure to PM_2.5_ increases by 1.05 person/km^2^ at the 80th percentile, and 1.88 person/km^2^ at the 95th percentile, respectively.

## 1. Introduction

Fine particulate matter (PM_2.5_) has become the primary pollutant of air pollution in China [1]. Evidence has shown that long-term exposure to PM_2.5_, even at concentrations common to US urban areas, leads to increased risk of mortality [2,3] and cardiovascular disease [4,5]. In addition, PM_2.5_ also impacts atmospheric visibility [6] and regional climate [7]. So, public opinion has been paying more and more attention to PM_2.5_ pollution. As a result, many researches were performed to analyze the characteristics, sources, and chemical compositions of PM_2.5_ based on site monitoring. For example, many mega-cities and heavily polluted regions (e.g., Beijing [8,9,10], Shanghai [11], Nanjing [12]) and urban agglomerations (e.g., the Jing-Jin-Ji region [13,14], Yangtze River delta [15], Pearl River delta [16], and Chang-Zhu-Tan region [17]) in China have been investigated. Other studies explored the spatiotemporal characteristics of PM_2.5_ in the whole of China based on remote sensing data [18,19], indicating that the spatial distribution of annual mean PM_2.5_ concentration coincides with China’s three gradient terrains. Besides, there are several studies based on specific surveys [20,21], a spatially aggregated level on specific regions [22,23]. Lin et al. [24] studied the spatiotemporal variation of PM_2.5_ and its relationship with geographic and socioeconomic factors in China based on PM_2.5_ concentration dataset released by the Center for International Earth Science Information Network (CIESIN)/Columbia University [25]. The results showed that high PM_2.5_ concentrations are mostly found in regions with high populations and rapid urban expansion in China for years of 2001–2010 [24].

Population exposure is often used to quantify the adverse health impacts of regional environment pollution. The variations of population exposure to PM_2.5_ across mega-cities [26], typical urban agglomerations [27], and mainland China [28] have been analyzed with gridded satellite retrievals or interpolated PM_2.5_ data. Shen and Yao [27] compared the correlation coefficient between population exposure to PM_2.5_ on the grid-level and the city-level, indicating the existence of the spatial heterogeneity of the relationship. So, the spatial scale seems to be a fundamental factor which cannot be ignored in creating and analyzing the relationship between environmental exposure and socio-economic factors [29].

The geographically weighted regression (GWR) model is commonly used to detect the spatial relationships between environmental and socioeconomic factors (or other ones) [24,30]. The GWR model is developed to explore the spatial heterogeneity, producing a set of local estimates of the parameters which demonstrate the spatial inhomogeneity of the relationship. This method cannot give the quantitative assessment of the relationship, although it can determine whether the relationship is positive or negative.

The objective of this study is to determine the appropriate spatial scale for analysis and then to conduct quantitative evaluation of the connection between population exposure to PM_2.5_ pollution and socio-economic factors. Three kinds of gridded data with 1 km spatial resolution were used, which are annual average PM_2.5_ concentration, population, and Gross Domestic Product (GDP) in mainland China for 2014. Three different methods were implemented for analysis, which are spatial correlation analysis, cumulative percent distribution, and quantile regression. The above approach is more likely to deeply understand the quantitative impact of socio-economic factors on population exposure to PM_2.5_. It is hoped that these analyses can provide a meaningful reference for decision making in the process of urbanization.

The remainder of this paper is organized as follows: a brief description of the data sources and methodologies is given in Section 2. Then in Section 3.1 the spatial distributions of population exposure and economic effects on PM_2.5_ over mainland China in 2014 are demonstrated. Section 3.2 discusses the spatial correlation between population exposure to socio-economic factors, and the optimal spatial scale and a further quantitative assessment is given in Section 3.3. Finally, the conclusions are drawn in Section 4.

## 2. Data and Methods

### 2.1. Datasets

**Figure 1:**
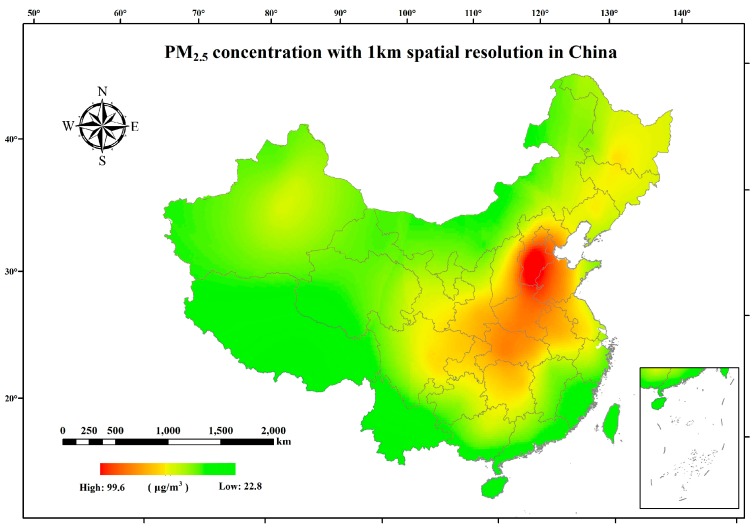
The spatial distribution of PM_2.5_ (suspended particles with aerodynamic diameter less that 2.5 μm) concentration in China in 2014.

The China National Environmental Monitoring Center (CNEMC) has been providing hourly PM_2.5_ observations in China since 1 January 2013 [31]. The systematic air quality monitoring network was composed of approximately 1497 monitoring sites by the end of 2014 [27]. In this study, the annual mean PM_2.5_ concentration was calculated at each site by averaging the hourly observations from 1 January to 31 December in 2014 (with the absence rate less than 1%). Furthermore, a co-krigin method was introduced to estimate grid-level PM_2.5_ concentration (1 × 1 km) based on site observations and digital elevation model (DEM) data. The gridded DEM data with a spatial resolution of approximately 90 m was extracted from the Shuttle Radar Topography Mission (SRTM) digital elevation product released by the National Aeronautics and Space Administration (NASA). Figure 1 shows the spatial distribution of annual PM_2.5_ concentration in 2014. It can be seen that mid-eastern China suffers more serious PM_2.5_ pollution than other areas (over 90 μg/m^3^), especially in the southern area of Hebei province, and this pattern has persisted for several years [18].

**Figure 2:**
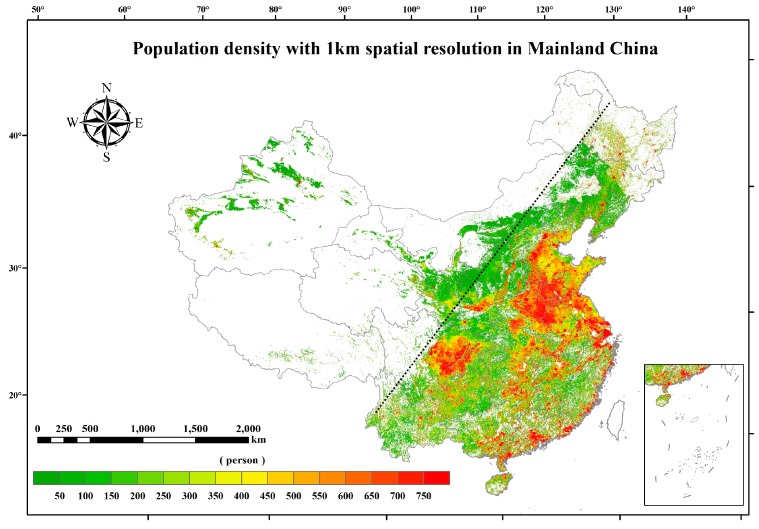
Gridded population density in mainland China in 2014 with a spatial resolution of 1 km. The black dotted line represents the “Heihe-Tengchong Line”.

**Figure 3:**
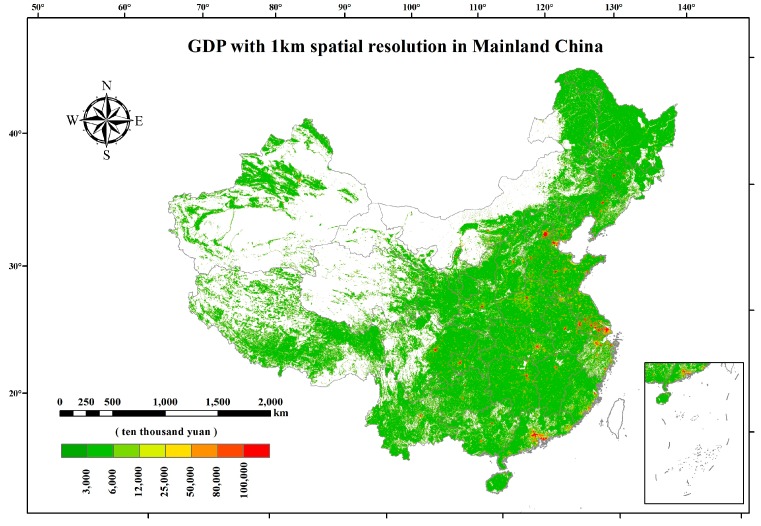
Gridded Gross Domestic Product (GDP) in mainland China in 2014 with a spatial resolution of 1 km. Blank in the figure means areas without GDP.

Gridded population and GDP data were provided by the National Earth System Science Data Sharing Infrastructure. They were transformed from census data with a spatial resolution of 1 km, considering the relationship among demographical, GDP, and land use types, and were adjusted with nighttime lights data derived from National Oceanic and Atmospheric Administration NOAA’s National Center for Environmental Information (NCEI) [24]. It can be seen from Figure 2 that the population distribution in mainland China in 2014 was divided into two parts by the “Heihe-Tengchong Line” (also known internationally as the Hu line), which is a geo-demographic demarcation line proposed by Hu [32]. Most of the Chinese people live in the eastern region marked by this line. Figure 3 shows the GDP distribution in mainland China in 2014. Each provincial capital contributed much more GDP than other cities in all provinces.

In this study, population data were applied to calculate the population exposure of PM_2.5_, while GDP data were used to characterize the economic development level of China. Because of the lack of population and GDP data from Hongkong, Macau, and Taiwai, the following analyses were carried out only in mainland China. Then, the population exposure and its relationship with socio-economic factors were analyzed at three spatial scales, which were grid level, county level, and city level.

### 2.2. Population Exposure Calculation

Population exposure (PE) is often used as an indicator of exposure assessment. If there is no population, there is no exposure [33]. In this paper, the population exposure to PM_2.5_ was illustrated at three different spatial scales, which were grid-level, county-level, and city-level. Grid-level population exposure to PM_2.5_ can be calculated as,

(1) $$PE_{i}=P_{i}C_{i}$$

where *i* stands for each grid cell; *PE_i_* represents the population exposure at gird *i*; *P_i_* is the population density; and *C_i_* is the PM_2.5_ concentration.

County-level and city-level population exposure to PM_2.5_ are calculated with the zonal statistics method based on grid-level PE. These statistics were performed using the software environment ArcGIS and the Zonal Toolset (version 10.2; http://resources.arcgis.com/en/help/main/10.2/).

### 2.3. Spatial Correlation Analysis

On the basis of grid-level PM_2.5_ concentration and socio-economic data, the band collection statistics method was introduced to acquire the general correlation between PM_2.5_ concentration and socio-economic factors. The relationships can be depicted with a correlation matrix, which is a measure of dependency between the factors.

First, the covariance between bands *i* and *j* can be determined by the following formula,

(2) $$Cov_{ij}=\frac{\sum_{k=1}^{N}\left(Z_{ik}-\mu_{i}\right)\left(Z_{jk}-\mu_{j}\right)}{N-1}$$

where *Cov_ij_* represents covariance between bands *i* and *j*; *Z* is the value of a given grid cell; *i, j* are bands of a stack (e.g., GDP and PE); *μ* stands for the mean value of a band; *N* is the number of grid cells; *k* denotes a particular grid cell.

Then, the equation to calculate the correlation is as follows,

(3) $$Corr_{ij}=\frac{Cov_{ij}}{\sqrt{Var_{i}}\sqrt{Var_{j}}}$$

where *Cov_ij_* represents covariance between bands *i* and *j*; $\sqrt{Var_{i}}\;\mathrm{and}\;\sqrt{Var_{j}}$ are standard deviations of the given bands. The calculated correlation ranges from −1 to +1, indicating whether the correlation is positive or negative. The magnitudes of the covariance matrix are dependent on units, while the ones of the correlation matrix are not.

### 2.4. Quantile Regression Method

In this research, the quantile regression method was applied to the further analysis of economic effects on PM_2.5_. Unlike ordinary linear regression, quantile regression essentially transforms a conditional distribution function into a conditional quantile function of the response variable by slicing it into segments [34], and is not based on parametric assumptions regarding specificities of the underlying data distributions. In ordinary linear regression, the conditional mean of a response random variable *Y* is modelled as linearly related to a random variable *X*, which is,

(4) $$E\left[Y|X\right]=\beta X+\gamma =f_{\left(\left(\beta ,\gamma \right)\right)}\left(X\right)$$

where β denotes the slope and γ is the intercept. They are estimated by minimizing the sum of the squared residuals for a realization (*x*, *y*) of (*X*, *Y*).

(5) $$\left(\beta ,\gamma \right)=argmin\left(\beta^{\prime },\gamma^{\prime }\right)\underset{i}{\sum }{\left(y_{i}-f_{\left(\beta^{\prime },\gamma^{\prime }\right)}\left(x_{i}\right)\right)}^{2}$$

In the case of quantile regression, E[Y|X] is instead by a quantile of the response variable *Y* conditional on *X*, $Q_{\tau }\left[Y|X\right]$. For each quantile *τ* ∈ [0, 1], the linear quantile regression can be described as,

(6) $$Q_{\tau }\left[Y|X\right]=f_{\left(\left(\beta_{\tau },\gamma_{\tau }\right)\right)}\left(X\right)$$

for a (*x*, *y*) the slope $\beta_{\tau }$ and intercept $\gamma_{\tau }$ are obtained by minimizing the sum of the asymmetrically weighted absolute residuals,

(7) $$\left(\beta_{\tau },\gamma_{\tau }\right)=argmin\left(\beta_{\tau }{}^{\prime },\gamma_{\tau }{}^{\prime }\right)\underset{i}{\sum }\rho_{\tau }\left(y_{i}-f_{\left(\beta_{\tau }{}^{\prime },\gamma_{\tau }{}^{\prime }\right)}\left(x_{i}\right)\right)$$

with $\rho_{\tau }$ denoting the tilted absolute value function, which gives differing weights to residuals $r_{i}$ depending on the quantile under consideration [35], that is,

(8) $$\rho_{\tau }\left(r_{i}\right)=\{\begin{array}{c} \begin{array}{cc} \tau r_{i} & \;\;\;\;\;\;\;\;\;\;\;r_{i}\geq 0 \end{array}\\ \begin{array}{cc} \left(\tau -1\right)r_{i} & r_{i}<0 \end{array} \end{array}$$

## 3. Results and Discussion

### 3.1. Population Exposure and Economic Effects on PM_2.5_

**Figure 4:**
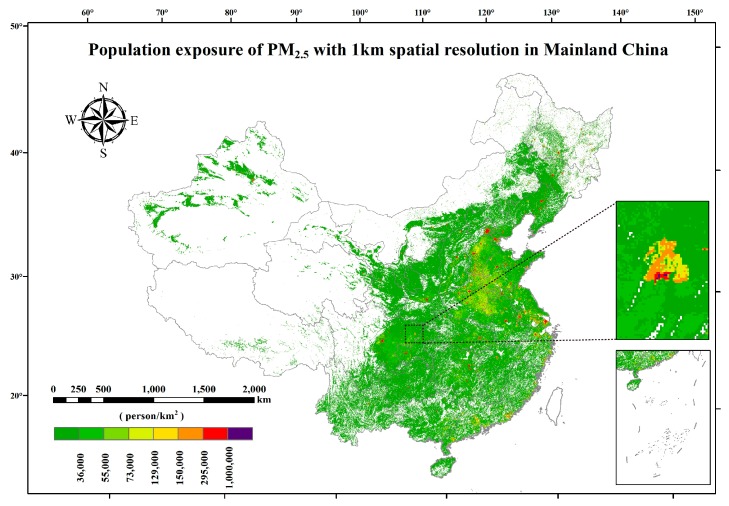
Gridded population exposure to PM_2.5_ in mainland China in 2014 with a spatial resolution of 1 km. Blank in the figure means areas without population exposure to PM_2.5_.

The annual average concentrations of PM_2.5_ was 49.6 μg/m^3^ over mainland China, which is approximately 5 times the air quality guidelines (AQG) set by The World Health Organization (WHO) of 10 μg/m^3^. The total population of mainland China in 2014 was about 1.368 billion. Figure 4 showed the spatial distribution of population exposure to PM_2.5_ in mainland China for 2014. It can be obviously seen that mega-cities often suffered higher population exposure, and the two mega-cities of Pearl River Delta (Guangzhou and Shenzhen) enjoyed much lower population exposure to PM_2.5_ than other provincial capitals.

However, it is found that the highest population exposure to PM_2.5_ appeared in a lesser-known city, Dazhou city, located in Sichuan province, rather than any other mega-cities or second-tier cities. This phenomenon was imputed to two factors. One reason was that industry pollution (e.g., steel, mining, fossil-fuel power, cement) was really heavy here in 2014, the other reason was the unfavorable topographic factor in this region. As a highly built-up and densely populated city surrounded by mountains on three sides, Dazhou city depends on strong winds to disperse air pollution. In recent years, as a result of drastic measures of environmental protection taken by the local government, the percentage of days with air quality indexes (AQIs) reaching defined standards in Dazhou city achieved 83.6% in 2017.

**Figure 5:**
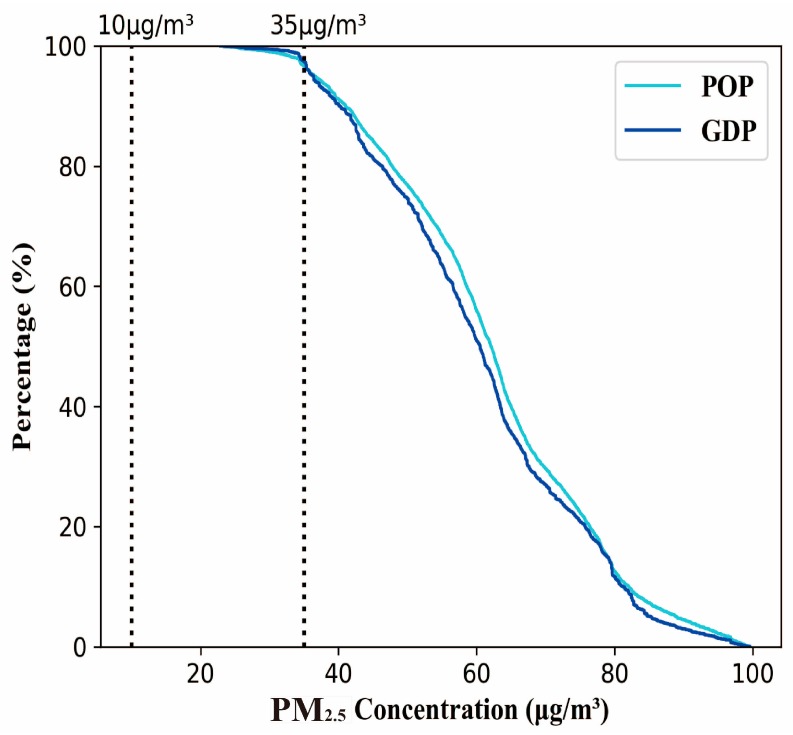
Cumulative distribution of annual mean PM_2.5_ in mainland China for 2014 refer to the WHO air quality guidelines (AQG) of 10, and 35 μg/m^3^. POP stands for population.

The cumulative percentage of the population and GDP (0–100%) in mainland China was calculated based on the grid-level data to express the frequency distribution of annual mean PM_2.5_ concentration (Figure 5). The results demonstrated that the WHO AQG (10 μg/m^3^) for PM_2.5_ was exceeded for 100% of the population in mainland China. An existing research indicated that there were about 70% population of East Asia living above the WHO Interim Target-1 of 35 μg/m^3^ [36]. It should be noted that the proportion of the population of mainland China living above this level was exceeded by 97%. There were even 58% of the population of mainland China living in a PM_2.5_ concentration of 60 μg/m^3^, while all populations in the three major urban agglomerations (Jing-Jin-Ji, the Yangtze River delta, and Sichuan-Chongqing region) lived under the WHO Interim Target-1 (35 μg/m^3^). Figure 5 also showed that only 2% of the GDP was produced in mainland China with annual mean PM_2.5_ concentration under the WHO Interim Target-1, while all GDP exceeded the WHO AQG of 10 μg/m^3^. There were more than half of the total GDP of mainland China generating within a PM_2.5_ concentration of 60 μg/m^3^, and 14% of the GDP producing above 80 μg/m^3^.

### 3.2. Spatial Correlation between PM_2.5_ and Socio-Economic Factors

In this section, the correlation between PM_2.5_ and socio-economic factors was examined with the band collection statistics method based on grid-level data. Table 1 showed the statistical results. All of the associations among the involved variables present a positive relation. The formula of population exposure can explicitly explain the weak relation with PM_2.5_ and the strong relation with population, considering the different orders of magnitude between PM_2.5_ concentration and population. The correlation coefficient between GDP and population (*R* = 0.74) indicates that people gather in the areas with high GDP in mainland China.

A previous study hypothesized that higher populations and GDP may cause higher PM_2.5_ concentrations [20]. However, as can be seen from Table 1, the correlation coefficients for population, GDP, and population exposure to PM_2.5_ are 0.07, 0.19, and 0.3, respectively, which indicates that they have a weak correlation with PM_2.5_ in mainland China for 2014. In contrast, an obviously positive correlation between GDP and population exposure to PM_2.5_ with a correlation coefficient of 0.88 is observed, which is statistically significant. Another study revealed the similar results in the four typical urban agglomerations of China [22]. However, the magnitude of correlation coefficients cannot quantify the influence among variables directly [14], but just provides a valuable hint for the following quantile regression analysis between GDP and population exposure to PM_2.5_.

### 3.3. Quantile Regression Analysis of Economic Effects on PM_2.5_ Exposure

**Figure 6:**
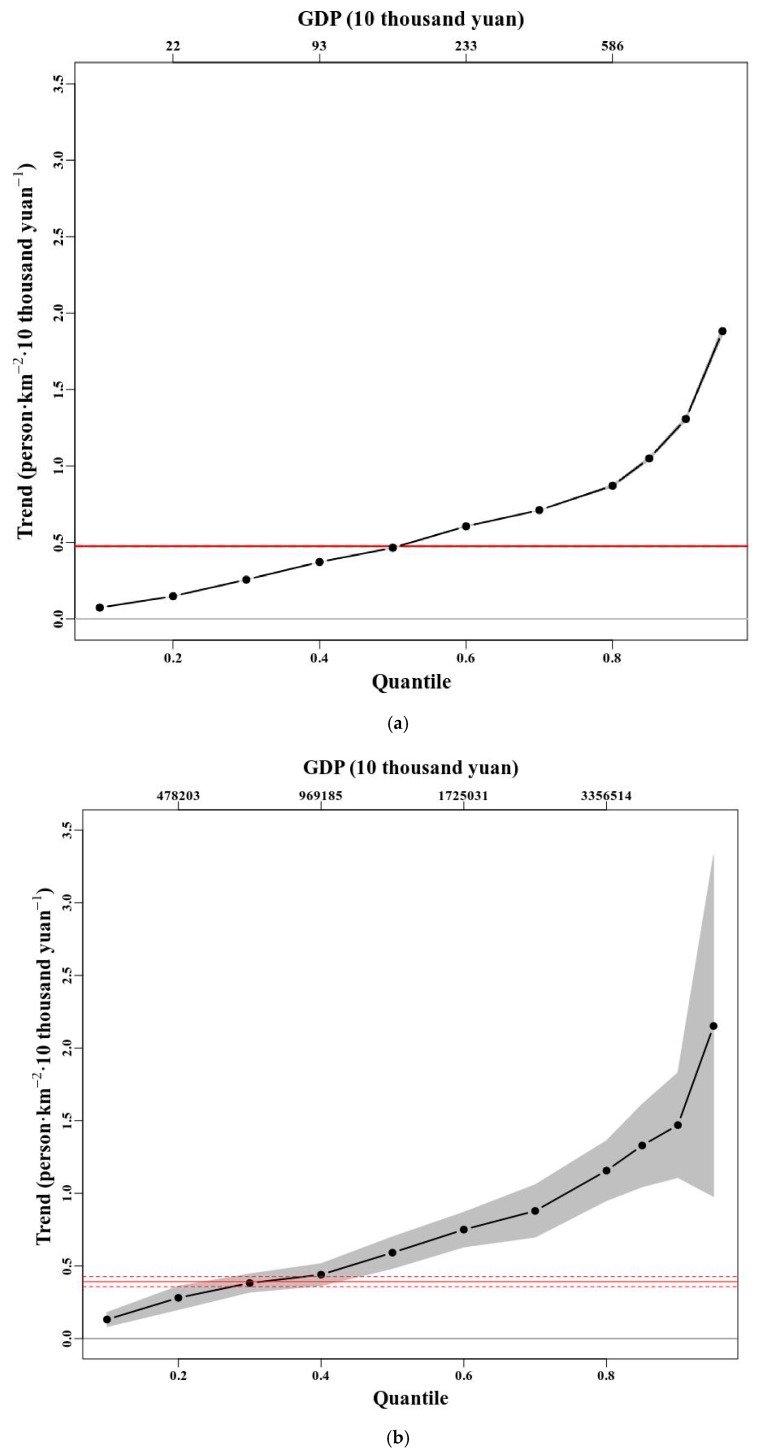

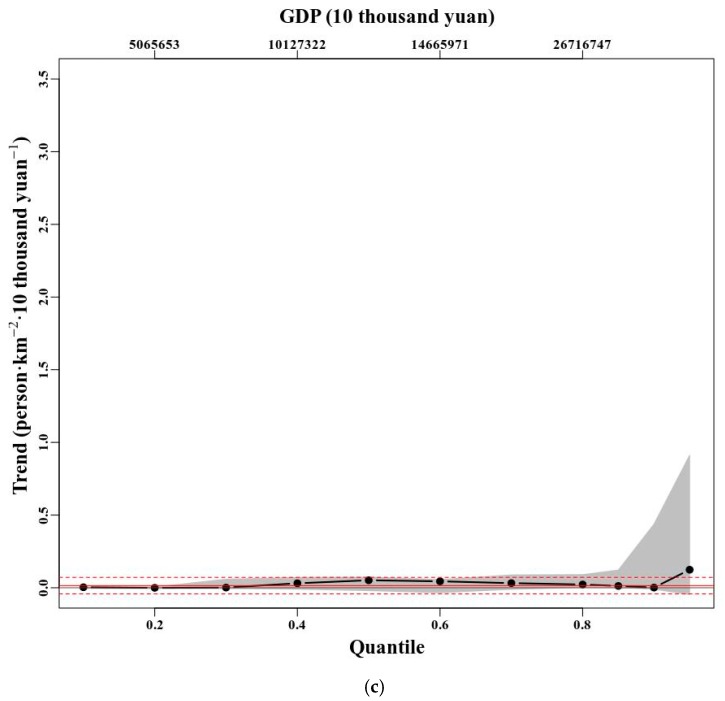
(**a**) Quantile regression slopes of the 0.1–0.95 quantiles of GDP in relation to population exposure to PM_2.5_ on the grid-level spatial scale. (**b**) Quantile regression slopes of the 0.1–0.95 quantiles of GDP in relation to population exposure to PM_2.5_ on the county-level spatial scales. (**c**) Quantile regression slopes of the 0.1–0.95 quantiles of GDP in relation to population exposure to PM_2.5_ on the city-level spatial scale. Upper quantiles are displayed with smaller step length (such as 0.85, 0.9, 0.95).

To quantify how population exposure to PM_2.5_ is affected by GDP, Figure 6 showed the respective quantile regression slopes. A quantile is a point taken from the inverse cumulative distribution function of the set of GDP so that, for example, the 0.8 quantile is the value such that 80% of the GDP samples are below this value (80th percentile). The value of GDP over the entire dataset corresponding to the selected quantiles are also displayed in Figure 6. For the observations, the 95% confidence intervals of the estimated slopes are also shown as shading, under the assumption that the errors are independent and identically distributed. Significant slopes (5% significance level, two-tailed test) are highlighted with bold dots. For comparison, the solid red lines are from a least-squares regression of GDP as a function of population exposure and the dashed red lines delineate the 95% point-wise confidence band about this trend. These analyses are shown at three spatial scales (based on the gridded data, county-level, and city-level data).

As in Figure 6a,b, trends significantly above zero are seen for all quantiles. Gradually increasing positive slopes for increasing GDP and population exposure to PM_2.5_ quantiles are identified from the datasets. The strong relation of upper quantiles of GDP with population exposure to PM_2.5_ is found to be a robust feature on both grid-level (Figure 6a) and county-level (Figure 6b) spatial scales. The upward trends are similar for the grid-level and county-level, the trend raised from 0.13 to 2.15 on the county-level, while the trend rose from 0.07 to 1.88 on the grid-level. However, the confidence intervals showed that the inferred slopes were slightly more pronounced and significant for the grid-level. The best estimates on both the grid-level and the county-level indicated that the highest population exposure to PM_2.5_ were getting higher with increasing GDP, but the ranges of uncertainty were relatively large on the county-level.

In contrast, a weak relationship between GDP and population exposure to PM_2.5_ quantiles is identified, which is generally insignificant at the city-level spatial scale (Figure 6c). At this scale, the relations of the quantiles of GDP with population exposure to PM_2.5_ do not exhibit a clear tendency with increasing quantiles (*p* > 0.1 for almost all quantiles). It illustrated that the spatial variation of population exposure and GDP was ignored to some extent at the larger scale, and the width of the confidence intervals provided an evidence that analysis on the grid-level seemed to be the optimal spatial scale while investigating the economic effects. Thus, the spatial scale effect indeed seems critical for explaining the identified relationship between GDP and population exposure to PM_2.5_ in mainland China. Moreover, the results indicated that the quality of the gridded population and GDP data used in this research, which were adjusted with the nighttime lights data, can meet the precision requirements for data analysis.

For further analysis, trends, associated standard errors and *p* values for upper-quantile (≥85th-percentile) GDP as a function of population exposure to PM_2.5_ are displayed in Table 2. Sample size (number of samples) is given in parentheses next to the spatial scale level. Values are shown for selected upper quantiles (0.80, 0.85, 0.90, and 0.95). For each quantile, *Trend* denotes the inferred slopes at all spatial scales in the analysis. We noted significant (*p* < 0.01) trend increases for all quantile levels, and upward trends at all spatial scales for the highest quantile considered (95th percentile), although not all trends at this extreme quantile are statistically significant (insignificant on the city-level). For a 10 thousand yuan rise in GDP, the results showed an increase of 1.05, 1.33 person/km^2^ in the value of the 80th percentile and 1.88, 2.15 person/km^2^ in the value of the 95th percentile, respectively, on the grid-level and the county-level. This means that the economic growth in areas with high GDP in China is at the cost of the heavier population exposure, which is typical of the extensive economic growth.

## 4. Conclusions

In this study, spatial characteristics in mainland China for 2014 were evaluated based on the gridded PM_2.5_ concentration, population, and GDP data with 1 km spatial resolution. The economic effects on PM_2.5_ were investigated by cumulative percent distribution, as well as spatial correlation coefficients, and economic effects on population exposure to PM_2.5_ were estimated with the quantile regression method at three spatial scales. The main findings were as follows:

(1) Quantile regression demonstrated that the highest population exposure to PM_2.5_ was rising with increasing GDP in mainland China for 2014. The tiny uncertainty on the grid-level suggested the optimal spatial scale for socio-economic effects analysis.

(2) A violent upward trend of population exposure to PM_2.5_ appeared at the 80th percentile GDP. For a 10 thousand yuan rise in GDP, an increase in population exposure to PM_2.5_ of 1.05 person/km^2^, 1.88 person/km^2^ in the value of the 80th percentile and the extreme value (95th percentile) GDP, respectively, on the grid-level spatial scale.

(3) Population exposure to PM_2.5_ was commonly higher in mega-cities in mainland China. However, a lesser known city named Dazhou suffered the highest population exposure to PM_2.5_ for 2014, as a result of its pollution from industry and unfavorable terrain.

This study presents the quantitative assessment of the relationship between GDP and population exposure to PM_2.5_ from a new perspective. In future research, a time series analysis will be performed to acquire a deeper understanding of the complex effects between air quality, socio-economic effects, and public health.

## Figures and Tables

**Table 1:** The correlation matrix of the band collection statistics.

Variable	PM_2.5_	Population	GDP	Population Exposure
PM_2.5_	-	0.07 *	0.18 *	0.3 *
Population	0.07 *	-	0.73 *	0.66 *
GDP	0.19 *	0.74 *	-	0.88 *
Population Exposure	0.3 *	0.66 *	0.88 *	-

Notes: * *p* < 0.05. All results have statistical significance.

**Table 2:** Summary statistics results of upper-quantile (≥80th-percentile) GDP (including trends, standard errors, and *p* values) as a function of population exposure to PM_2.5_.

Statistic	Quantile			
	80%	85%	90%	95%
Grid (2,759,981 samples)				
GDP (10 thousand yuan)	585.89	906.79	1678.91	5246.21
Trend (person km^−2^ 10 thousand yuan^−1^)	0.87 *	1.05 *	1.31 *	1.88 *
Std. Error	0.005	0.0076	0.009	0.018
*p*	<0.001	<0.001	<0.001	<0.001
County (2375 samples)				
GDP (10 thousand yuan)	3,356,513.64	4,397,744.36	6,241,262.98	9,844,463.44
Trend (person km^−2^ 10 thousand yuan^−1^)	1.16 *	1.33 *	1.47 *	2.15 *
Std. Error	0.125	0.173	0.22	0.71
*p*	<0.001	<0.001	<0.001	0.003
City (349 samples)				
GDP (10 thousand yuan)	26,716,747.01	32,623,702.94	46,608,724.75	69,396,042.94
Trend (person km^−2^ 10 thousand yuan^−1^)	0.022	0.012	0.001	0.124
Std. Error	0.018	0.017	0.12	0.275
*p*	0.225	0.48	0.99	0.65

Notes: * *p* < 0.05. Trend without * is not significant.
